# The Neglected Microbial Components of Commercial Probiotic Formulations

**DOI:** 10.3390/microorganisms8081177

**Published:** 2020-08-03

**Authors:** Walter Fiore, Stefania Arioli, Simone Guglielmetti

**Affiliations:** Division of Food Microbiology and Bioprocesses, Department of Food, Environmental and Nutritional Sciences, University of Milan, 20133 Milan, Italy; walter.fiore@unimi.it (W.F.); stefania.arioli@unimi.it (S.A.)

**Keywords:** paraprobiotics, postbiotics, flow cytometry, cell viability, food supplements

## Abstract

Producers of probiotic products are legally required to indicate on the label only the minimum numbers of viable microorganisms at the end of shelf life expressed as colony-forming units (CFUs). Label specifications, however, describe only a fraction of the actual microbiological content of a probiotic formulation. This paper describes the microbiological components of a probiotic product that are not mentioned on the label, such as the actual number of CFUs, the presence of viable cells that cannot generate colonies on agar plates, and the abundance of dead cells. These “hidden” microbial fractions in probiotic products, the abundance of which may change during the shelf life, can promote biological responses in the host. Therefore, they should not be ignored because they may influence the efficacy and can be relevant for immunocompromised or fragile consumers. In conclusion, we propose the minimum requirements for microbiological characterization of probiotic products to be adopted for label specifications and clinical studies.

## 1. Introduction

Probiotics are “live microorganisms that, when administered in adequate amounts, confer a health benefit on the host” [1]. Therefore, by definition, the term probiotic is restricted to live microbial cells. The number of live microbes in a probiotic formulation is generally recognized as being relevant for the effectiveness of the product. Accordingly, a review of the literature led national public authorities in Canada and Italy to suggest in their guidelines that the minimum number of live probiotic microorganisms imparting general health benefits should be at least 10^9^ colony-forming units (CFUs) per day (Italian Ministry of Health) or per serving (Health Canada) [1]. In addition, reportedly, for specific health conditions, benefits can only be observed at above a certain concentration of live cells of the probiotic; for instance, *Lactobacillus rhamnosus* GG was shown to most likely be effective in treating infectious diarrhea in children when administered at a dose of at least 10^10^ CFUs per day [2,3]. In another study, the authors observed that probiotics may significantly reduce systolic and diastolic blood pressure only when daily doses ≥10^11^ CFUs were used [4].

Since the amount of live microbial cells administered is profoundly relevant for the health benefit that probiotics can provide to hosts, the regulations of several countries specify that the minimum numbers of viable microorganisms at the end of the shelf life must be indicated on the label of the probiotic product. This is the only information that the producers of probiotic products are legally required to indicate on the label. Nonetheless, this label specification describes only a fraction of the actual microbiological content of a probiotic formulation. Within this framework, in this opinion paper, we will describe the microbiological components constituting a probiotic product that are not mentioned on the label, discussing their relevance in the context of clinical research aiming to elucidate the health-promoting properties of probiotics. Finally, we will discuss methodological considerations in support of the use of flow cytometry (FC) for the characterization of microbial viability in a probiotic formulation.

## 2. “Overfilling” of Viable Microbial Cells during Probiotic Product Manufacturing

Producers constantly evaluate the viable microbial content of commercial probiotic products to ensure compliance with the information reported on the label. Specifically, the amount of microbial CFUs per single dose indicated on the label refers to the minimum concentration of live cells that must be present at the end of the shelf life. During the shelf life of any probiotic product, which is typically longer than 12 months, some of the microbial cells inevitably die. To address this issue, excess CFUs of probiotic microorganisms are commonly added to the product (i.e., “overfilling” of capsules, tablets, or sachets with additional microbial cells) to guarantee that the viable concentration does not decrease below the limit declared on the label before the end of the shelf life [5]. However, overfilling leads to a significant increase in production costs. For this reason, probiotic producers try to limit the cost as much as possible by adopting and optimizing strategies aimed at preserving microbial survival, which involve the cultivation media used for the production of the biomass, the freeze-drying protocols, the protective agents used during freeze-drying, the microencapsulation technique, and the packaging systems [6,7,8,9,10]. According to our experience of collaboration with companies that produces probiotic formulations for the European market, cell overfilling can range between 1.5 and 4 times, depending on the microorganism, dosage form and packaging (Fiore, Arioli, and Guglielmetti, personal communication).

In conclusion, the overfilling of probiotic products with excess viable microbial cells is practically always adopted, resulting in a fraction of CFUs that is not declared on the label but can surely contribute to the health effects of the product. Therefore, overfilling can significantly alter the actual load of a dose and, potentially, the outcomes of an intervention trial.

## 3. Viable Cells in Nongrowing States (the “Hidden” Viable Biomass)

By a convention that dates to the time of Koch, a microbial cell is considered “viable” if it reproduces to form a colony on an agar plate that supplies key nutrients for its replication. More recent advances, however, revealed that microorganisms may exist in a variety of metabolic states [11,12], most of which do not involve active replication [13]. In fact, in addition to “culturable” microbial cells, which may multiply and form a colony on agar plates, other non-colony-forming physiological states have been described, such as the “non-replicating” state, characterized by an active physiology and intact cytoplasmic membrane; the “starving” state, characterized by a dramatic decrease in metabolism; the “dormant” state, possessing low metabolic activity and an inability to divide without a preceding resuscitation phase (also defined as “viable but not culturable”; VBNC); and the “irreparably damaged” state, characterized by progressively declining metabolism that irreversibly leads to death [13]. The relevance of nonculturable states in the interaction with host health has been demonstrated for several pathogens, which were observed to retain their pathogenicity after entering the VBNC or dormant state [14,15,16]. Although rarely investigated, it has also been shown in probiotics that microbial cells that fail to grow on agar media can have several typical properties of viable cells, such as enzymatic activities (e.g., esterase and reductase activities) and an intact cytoplasmic membrane maintaining the electrochemical gradient [17,18,19]. Notably, viable unculturable microbial cells can still maintain some metabolic activity that could contribute to the promotion of health benefits [20]. In addition, plausibly, as also observed for intestinal pathogenic bacteria [21], probiotic microbial cells in nongrowing states may resuscitate (i.e., reacquire the ability to reproduce) once in the favorable environmental conditions of the gut, thereby starting to interact with host and resident microbiota similarly to cultivable viable bacteria.

## 4. Non-Viable Microbial Cells in Probiotic Formulations

The death of a bacterial cell is generally defined as “the point where the extent of injury is beyond the ability of a cell to resume growth” [22]. From a structural and functional point of view, non-viable (dead) microbial cells are characterized by an inability to reproduce, an irremediably damaged plasma membrane, a dissipated proton gradient, and an absence of any metabolic activity [23].

**Figure 1:**
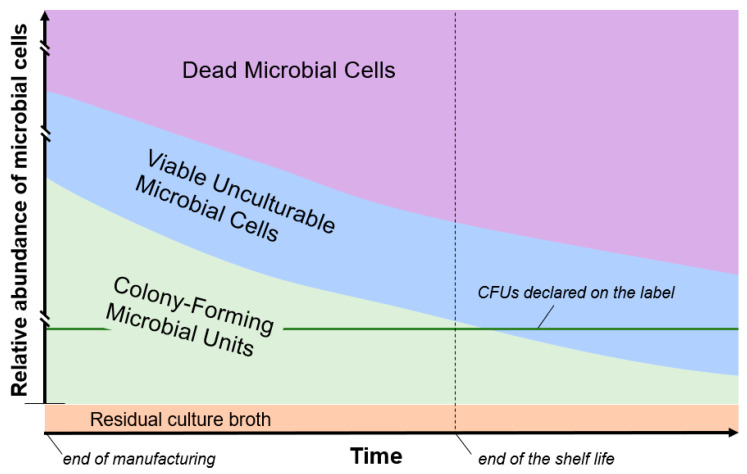
Exemplifying representation of the microbial viability states of a hypothetical probiotic product over time. The relative abundance of viable cells (green) progressively decreases over time, whereas that of dead cells (violet) progressively increases. Conversely, viable unculturable microbial cells (light blue) can potentially both increase and decrease over time. Culture broth residues from the manufacturing process are also present in the final formulation (orange). The real kinetics of the change in relative abundances is not represented.

Dead cells are virtually always present in a probiotic formulation. Some of these cells are generated by the stresses of industrial manufacturing processes, including biomass production and concentration, cryopreservation, and lyophilization [24]. In addition, a progressive increase in dead cells occurs during the shelf life according to cell death kinetics, which depend on taxon/strain-associated properties, product formulation and packaging, and storage conditions (temperature, darkness, controlled water activity) [25] (Figure 1).

By definition, viability is an essential prerequisite to qualify a microorganism as probiotic, and it is conventionally considered to be essential for the exertion of health benefits. In this regard, the first report of the Joint FAO/WHO Expert Consultation on Evaluation of Health and Nutritional Properties of Probiotics in Food in 2001 stated that “the ability to remain viable at the target site should be verified for each potential strain” [26]. Nonetheless, although viable probiotic cells may be more effective than the same non-viable microorganisms [27], increasing literature has demonstrated that inactivated (dead) probiotic cells may also exert beneficial effects on human health [28,29,30,31]. Accordingly, several products intentionally constituted of non-viable microbial cells are increasingly becoming available on the market [20,29,32,33,34,35]. For such products, the term “paraprobiotics” was proposed with the following specific definition: “non-viable microbial cells (intact or broken) or crude cell extracts (i.e., with complex chemical composition), which, when administered (orally or topically) in adequate amounts, confer a benefit on the human or animal consumer” [28]. The nonvital microbial material that falls within the definition of a paraprobiotic is able to interact with host health primarily through mechanisms of immunomodulation [28], mediated at the intestinal level (principally in the ileum; [36]) by the microbe-associated molecular patterns (MAMPs) that constitute the microbial cell, such as outer surface molecules [(lipo)teichoic acids [37], S-layer proteins [38], polysaccharidic capsules [39], outer surface proteins [40,41]] and other cellular components (e.g., genomic DNA and unmethylated cytosine-phosphate-guanine-containing oligodeoxynucleotides [42]).

## 5. Extracellular Microbial Products in Probiotic Formulations

Usually, in industrial manufacturing processes, immediately after fermentation, the broth culture is concentrated (often from 1:5 to 1:10) by continuous centrifugation before freeze-drying. Therefore, the lyophilized microbial biomass used to prepare a probiotic product contains the residual growth medium, which includes microbial metabolites produced and secreted/released by probiotic cells during growth. Such microbial products may include primary metabolites (e.g., lactate, acetate, propionate), bacteriocins, reuterin, or other secreted molecules (e.g., immunomodulatory secreted peptides; [43]), which can be defined as “postbiotics” [44]. The actual contribution of these molecules to the interaction of probiotic formulations on host health is not known but, in light of data obtained from in vitro experiments, cannot be excluded [45,46,47].

## 6. Methodologies for the Microbiological Characterization of Probiotic Products

The analysis of a probiotic formulation is conventionally carried out by counting CFUs through the serial dilution method to measure the viable cell number. This method allows only the identification of microbial units (single cells or aggregates) that may create visible colonies on an agar medium, while being unable to provide any information on cells that are dead or present in unculturable physiological states. To obtain a more comprehensive microbiological characterization of probiotic products, FC has been proposed [48]. In fact, FC can overcome some disadvantages of the cell viability measurement based on plating, such as the impossibility of providing results in real time, the lack of information regarding cell integrity and metabolic activity, and, most importantly, the inability to quantify both dead cells and viable microbial cells that cannot produce colonies on agar plates. Specifically, FC associated with the use of fluorescent dyes can simultaneously generate data regarding viability, structural integrity, and physiological state in individual cells [49]. In addition, FC can analyze up to thousands of cells/events per second. For these reasons, FC is becoming increasingly popular as a rapid alternative method for detection, enumeration, and population profiling of microorganisms [50], including probiotics [18]. Accordingly, in 2015, the International Organization for Standardization and the International Dairy Federation (ISO and IDF) published a standard method for the quantification of active and/or total lactic acid bacteria and probiotics in dairy products and fermented milk products by FC (ISO19344:2015/IDF232:2015).

Notably, when FC is used for viability assessment of microorganisms in commercial probiotic products, cells in non-viable (injured, dead) states are always detected, increasing in relative abundance during storage [51] and frequently representing a dominant fraction of the probiotic formulation [48,52,53].

## 7. Conclusions

In this opinion paper, we discussed the microbiological components constituting a probiotic product, which can be more abundant than the number of live cells (expressed as CFUs) that the producers are legally required to indicate on the label, and include (i) additional CFUs, (ii) viable cells that cannot generate colonies on agar plates, and (iii) dead cells. Determining the complete composition in terms of physiological states of microbial cells in a probiotic product is difficult, and the abundance of the different microbiological components changes over time (Figure 1). Nonetheless, the use of FC can provide relevant contributions for comprehensive microbiological quantification of probiotic formulations.

In clinical trials aimed at studying the efficacy of a probiotic product, the quantity of CFUs indicated on the label is most often used as the reference value. However, ignoring the other microbial components of a probiotic product, since all these components (including dead cells) can promote biological responses in the host, can greatly restrict adequate comparison between different studies and different probiotic formulations. In addition, limiting the information on the label of a probiotic product to only the minimum concentration of CFUs guaranteed at the end of the shelf life significantly hampers the ability of health professionals to make a fully informed decision for the prescription of probiotic products. In fact, also the unculturable microbiological components (both viable and dead) reportedly influence the immune system. Therefore, overlooking these microbial fractions can represent a potential risk for immunocompromised or fragile subjects such as infants or older people.

Considering the above considerations, we believe that it is important to provide additional information about the microbial viability states of a probiotic product, both on the label and while conducting a clinical study. In this context, we propose the following minimum requirements for the microbiological characterization of a probiotic product:

- On the label of commercial products, in addition to the minimum number of CFUs at the end of the shelf life, the total number of microbial cells in the final product determined by FC at the end of manufacturing must be declared.
- In clinical trials, viable count by plating according to standard procedures (e.g., the methods reported in the document ISTISAN 08/36 by the Italian National Institute of Health) must be determined for each lot of the probiotic formulation under investigation immediately before the beginning of the intervention to determine the actual number of viable cells administered to study participants. In addition, the probiotic formulation must be analyzed by FC according to standard methods (e.g., ISO-IDF protocols) to determine the total numbers and proportions of viable, damaged, and dead microbial cells.

In our opinion, the minimum requirements for microbiological characterization proposed above can significantly contribute to a precise understanding of the potential efficacy of the myriad of probiotic products on the market in promoting health benefits.
